# Cholera outbreaks in sub-Saharan Africa during 2010-2019: a descriptive analysis

**DOI:** 10.1016/j.ijid.2022.05.039

**Published:** 2022-09

**Authors:** Qulu Zheng, Francisco J Luquero, Iza Ciglenecki, Joseph F Wamala, Abdinasir Abubakar, Placide Welo, Mukemil Hussen, Mesfin Wossen, Sebastian Yennan, Alama Keita, Justin Lessler, Andrew S Azman, Elizabeth C Lee

**Affiliations:** 1 Department of Epidemiology, Johns Hopkins Bloomberg School of Public Health, Baltimore, MD, USA; 2 Department of International Health, Johns Hopkins Bloomberg School of Public Health, Baltimore, MD, USA; 3 Global Alliance for Vaccines and Immunization (GAVI), Geneva, Switzerland; 4 Médecins Sans Frontières, Geneva, Switzerland; 5 World Health Organization, Juba, South Sudan; 6 WHO, Regional Office for Eastern Mediterranean, Cairo, Egypt; 7 PNECHOL-MD, Community IMCI, Ministry of Health, Democratic Republic of the Congo; 8 Disease and Health Events Surveillance and Response Directorate, Ethiopia Public Health Institute, Addis Ababa, Ethiopia; 9 Surveillance and Epidemiology, Nigeria Centre for Disease Control, Abuja, Nigeria; 10 Regional Office for West & Central Africa, UNICEF, Dakar, Senegal; 11 Department of Epidemiology, Gillings School of Global Public Health, University of North Carolina at Chapel Hill, Chapel Hill, NC, USA

**Keywords:** Cholera, Outbreaks, Sub-Saharan Africa

## Abstract

**Background:**

Cholera remains a public health threat but is inequitably distributed across sub-Saharan Africa. Lack of standardized reporting and inconsistent outbreak definitions limit our understanding of cholera outbreak epidemiology.

**Methods:**

From a database of cholera incidence and mortality, we extracted data from sub-Saharan Africa and reconstructed outbreaks of suspected cholera starting in January 2010 to December 2019 based on location-specific average weekly incidence rate thresholds. We then described the distribution of key outbreak metrics.

**Results:**

We identified 999 suspected cholera outbreaks in 744 regions across 25 sub-Saharan African countries. The outbreak periods accounted for 1.8 billion person-months (2% of the total during this period) from January 2010 to January 2020. Among 692 outbreaks reported from second-level administrative units (e.g., districts), the median attack rate was 0.8 per 1000 people (interquartile range (IQR), 0.3-2.4 per 1000), the median epidemic duration was 13 weeks (IQR, 8-19), and the median early outbreak reproductive number was 1.8 (range, 1.1-3.5). Larger attack rates were associated with longer times to outbreak peak, longer epidemic durations, and lower case fatality risks.

**Conclusions:**

This study provides a baseline from which the progress toward cholera control and essential statistics to inform outbreak management in sub-Saharan Africa can be monitored.

## Highlights

• Cholera outbreaks affected 2% of the sub-Saharan Africa populations in the 2010 decade.
• 692 outbreaks were identified in 492 districts with a systematic definition.
• Larger outbreaks were associated with longer durations and lower reported mortality.
• Population density was not always associated with severe cholera outbreak outcomes.
• Surveillance needs to be enhanced to improve cholera outbreak monitoring.

## Background

Cholera remains a public health threat worldwide, predominantly in countries with inadequate access to safe water and improved sanitation facilities. Sub-Saharan Africa is one of the regions with the highest cholera burden, where more than 140,000 suspected cases are estimated to occur each year in both endemic and epidemic settings (Lessler et al., 2018). From 2010 to 2019, 1,080,778 of the 4,426,844 (24%) cholera cases reported to the World Health Organization (WHO) came from sub-Saharan Africa (World Health Organization, 2020).

Large cholera outbreaks in refugee camps in the late 20th century have shaped public perception about the nature of epidemic cholera in sub-Saharan Africa (e.g., 1994 Goma outbreak among Rwandan refugees (Goma Epidemiology Group, 1995)); and data from these outbreaks continue to inform contemporary cholera outbreak responses (Global Task Force on Cholera Control, 2019). Ministries of health and humanitarian medical organizations, typical stakeholders in emergency cholera outbreak response, use summary statistics on historical cholera outbreaks to set expectations for the duration and magnitude of potential emergency responses (Global Task Force on Cholera Control, 2019; Olson et al., 2018; United Nations Children's Fund, 2013). As the distribution and density of populations in Africa have changed over the past few decades, so has water and sanitation infrastructure, and the dominant circulating pandemic *Vibrio cholerae* O1 lineages outbreak characteristics may have changed (Jones et al., 2020; Local Burden of Disease WaSH Collaborators, 2020; Weill et al., 2019). In addition, although many recent cholera outbreaks are associated with humanitarian crises, outbreaks in refugee and internally displaced people camps appear to be less prevalent and less explosive than in times past, likely because of improved camp coordination and preparedness, timely response to outbreaks, and increased preventive cholera vaccination in these settings (Shannon et al., 2019).

Over the past decade, reported cholera attack rates, case fatality risks (CFR), and estimates of the basic reproductive number have varied widely (Denue et al., 2018; Eisenberg et al., 2013; Gbary et al., 2011; Msyamboza et al., 2014; Mukandavire et al., 2011; Troeger et al., 2016). This heterogeneity in outbreak characteristics makes it hard to set clear expectations for how outbreaks may unfold. Previous studies of cholera outbreaks have focused on a narrow range of temporal and spatial scales, typically a single outbreak, so it is not often possible to examine factors associated with this heterogeneity (Denue et al., 2018; Eisenberg et al., 2013; Gbary et al., 2011; Msyamboza et al., 2014; Mukandavire et al., 2011; Troeger et al., 2016). Despite the existence of WHO outbreak definitions, the geographic range and time bounds of cholera outbreaks (e.g., the beginning and end) have been inconsistently defined in the past, further complicating the comparison of such statistics (World Health Organization, 2022).

Taking advantage of a large, global cholera incidence database, we performed a systematic examination of the characteristics and transmission dynamics of outbreaks of suspected cholera in sub-Saharan Africa from January 2010 through January 2020 (outbreaks that started within the 10-year time window of January 1, 2010, through December 31, 2019). These results are meant to provide a contemporary picture of cholera outbreaks in the region while serving as a practical resource in the control and management of cholera outbreaks in the years to come.

## Methods

### Cholera data

We extracted daily and weekly suspected cholera incidence data from the Global Task Force for Cholera Control's global cholera database, which contains public and confidential surveillance reports from sources including WHO, Médecins Sans Frontières (MSF), the Program for Monitoring Emerging Diseases, ReliefWeb, United Nations Children's Fund (UNICEF), ministries of health, and the scientific literature (Lessler et al., 2018; Moore et al., 2017). Typical reports were of suspected cholera cases using variants of the WHO-recommended suspected case definition (World Health Organization, 2022), though confirmed cases and deaths were also reported in select reports. Although this database is not comprehensive, it is, to our knowledge, the largest centralized source of global cholera incidence and mortality data. After aggregating daily incidence data to the weekly level and averaging reports from different sources that overlapped in space and time, we assembled weekly unified cholera incidence for each sub-national administrative unit (hence, “regions”). Further information on data coverage is described in Tables S1 and S2.

### Population data

Gridded, yearly population data (100m resolution) were obtained from WorldPop for each country associated with an outbreak (Linard et al., 2012; WorldPop, n.d.). Where shapefiles could be found from Humanitarian Data Exchange, Ministries of Health, Database of Global Administrative Areas, and Global Administrative Unit Layers (Database of Global Administrative Areas, n.d.; Humanitarian Data Exchange, 2022; GeoNetwork Team, n.d.), the outbreak region population was estimated using the gridded population data, and we considered the population in the year of the first outbreak day as the total population for the whole outbreak (Figure S1). We defined regions as “urban” when population density was equal to or higher than 1000 habitants per km^2^ and “rural” when below 1000 habitants per km^2^, following the previous convention (Chen et al., 2020; Hay et al., 2005).

### Outbreak definition

We applied a consistent operational outbreak definition across all regions in our analysis, which assumes that cholera outbreaks are defined by time periods where cholera incidence equals or exceeds the baseline incidence (also called the “outbreak threshold”) in a given region (Figure S2). An outbreak start was defined as a week when weekly cholera incidence reaches the outbreak threshold and is followed by increasing incidence for at least two consecutive weeks. For each unique region, the outbreak threshold was the average weekly cholera incidence between the first and last reported daily or weekly suspected cases; for this purpose, weeks without case reports were assumed to have no suspected cases. An outbreak was considered over after two consecutive weeks where weekly cholera incidence remained below the outbreak threshold, provided that this outbreak end was followed by a four-week “wash-out” period where weekly cholera incidence also remained below the outbreak threshold. Reported cases and mortality during the “wash-out” period were not considered part of the outbreak.

Outbreaks at sub-national administrative unit levels with uninterrupted reporting during the epidemic period were included in our main analysis (Figure S1). We removed outbreaks in the same region with overlapping time periods as duplicates, manually selecting those outbreaks with less censoring and fewer missing fields and observations. Outbreaks reported at different administrative reporting levels with overlapping time periods (e.g., province-level and district-level reporting) were counted as separate outbreaks and assessed independently in our study.

### Epidemic metrics

We calculated a standard set of metrics for each outbreak. *Attack rate* was defined as suspected cholera cases per 1000 population living in the outbreak region. CFR was defined as the ratio of cholera-associated deaths to suspected cholera cases in an outbreak. *Time to outbreak peak* was defined as the number of weeks between the start of an outbreak and the week with the most reported cases. We estimated instantaneous reproductive numbers using the EpiEstim package in R (Cori et al., 2013), where transmission was modeled in a Poisson process that calculates the incidence rate at a given time as the product of the instantaneous reproductive number (Rt) and average infectiousness. Using a Bayesian framework, we assigned a Gamma prior (mean = 2, SD = 0.7) to Rt and assumed the serial interval followed a Gamma distribution (mean = 4 days, SD = 3 days) and had a smoothing window of one week where transmission was assumed constant (Bi et al., 2018). The *early outbreak reproductive number* was the average of the instantaneous reproductive estimates over the first week of an outbreak (see details in Supplement) (Bi et al., 2018; Cori et al., 2013). We used the Wilcoxon rank sum test to test the null hypothesis that urban and rural settings had no differences in epidemic metrics. To further measure the associations between different epidemic metrics, Pearson bivariate correlation tests were performed for outbreaks reported across different administrative units (Best and Roberts, 1975; Hollander et al., 2013). For those epidemic metrics with a skewed distribution (i.e., outbreak attack rates, outbreak thresholds, and CFRs), a log transformation (i.e., log_10_-transformed scale) was used to normalize the covariate in correlation tests.

### Sensitivity analyses

Although we assumed zero suspected cases for weeks without case reports, many regions only report cases during officially declared outbreaks, leaving the possibility that more sporadic cases were not captured in the surveillance systems (Ajayi and Smith, 2019; Balakrish Nair and Takeda, 2014; Deen et al., 2020; Ganesan et al., 2020). Hence, setting the average of the reported weekly incidence as the fixed outbreak threshold may not reflect the true burden of cholera for a given region. To assess the sensitivity of the threshold and assumption, we repeated the analysis using a different definition for the outbreak threshold, which was defined as the average number of reported suspected cases per week during the first three epidemic weeks with an increasing number of reported cases (see Supplement).

Reported cases were aggregated to administrative units (e.g., district and province levels) in the surveillance systems, whereas the population of administrative units differed greatly within and between countries (Fontanelli et al., 2017). Therefore, outbreaks reported at the same spatial level may not be comparable to each other in terms of epidemic characteristics. To test other grouping methods, we further summarized the outbreak statistics by four total population size groups, including regions with a population of <10,000, regions with a population between 10,000-100,000, regions with a population between 100,000-1,000,000, and regions with a population of >1,000,000 (see Supplement).

### Code and data availability

All analyses were conducted in the R statistical programming language, version 4.0.2 (R Foundation for Statistical Computing). Outbreak extraction code and aggregated data unlinked from specific geographic locations are available on Github (https://github.com/HopkinsIDD/cholera_outbreaks_ssa).

## Results

**Figure 1:**
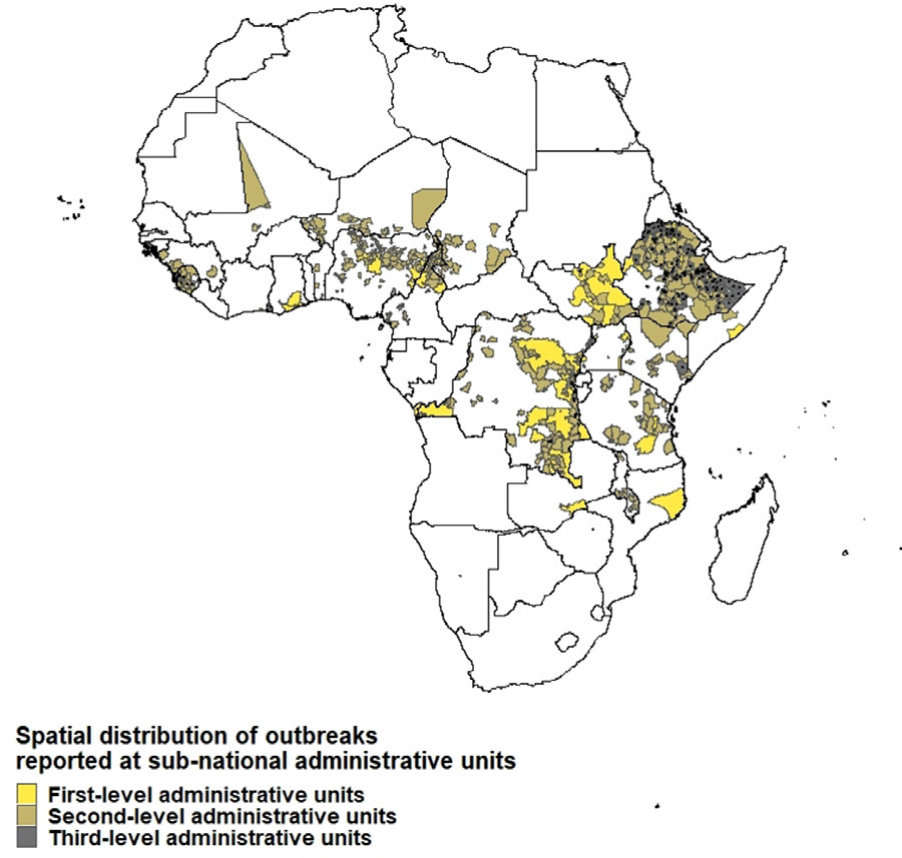
Spatial distribution of outbreaks reported at sub-national administrative units, January 2010 to January 2020. This map shows the regions that are associated with suspected cholera outbreaks. Different colors represent different sub-national administrative units at which cholera outbreaks were reported. Outbreaks in third-level administrative units are additionally marked with black dots to increase visibility on the map.

From January 2010 through January 2020, we captured 999 cholera outbreaks with 484,450 suspected cholera cases from 744 unique sub-national regions across 25 sub-Saharan African countries in our database. This included 62 outbreaks in 50 unique first-level administrative units, 692 outbreaks in 492 unique second-level administrative units, and 245 outbreaks in 202 unique third-level administrative units (Figures 1 and S1, Table S1). Among them, only 128 sub-national regions reported at least one confirmed cholera case, and 876 sub-national regions reported cholera-associated deaths.

**Figure 2:**
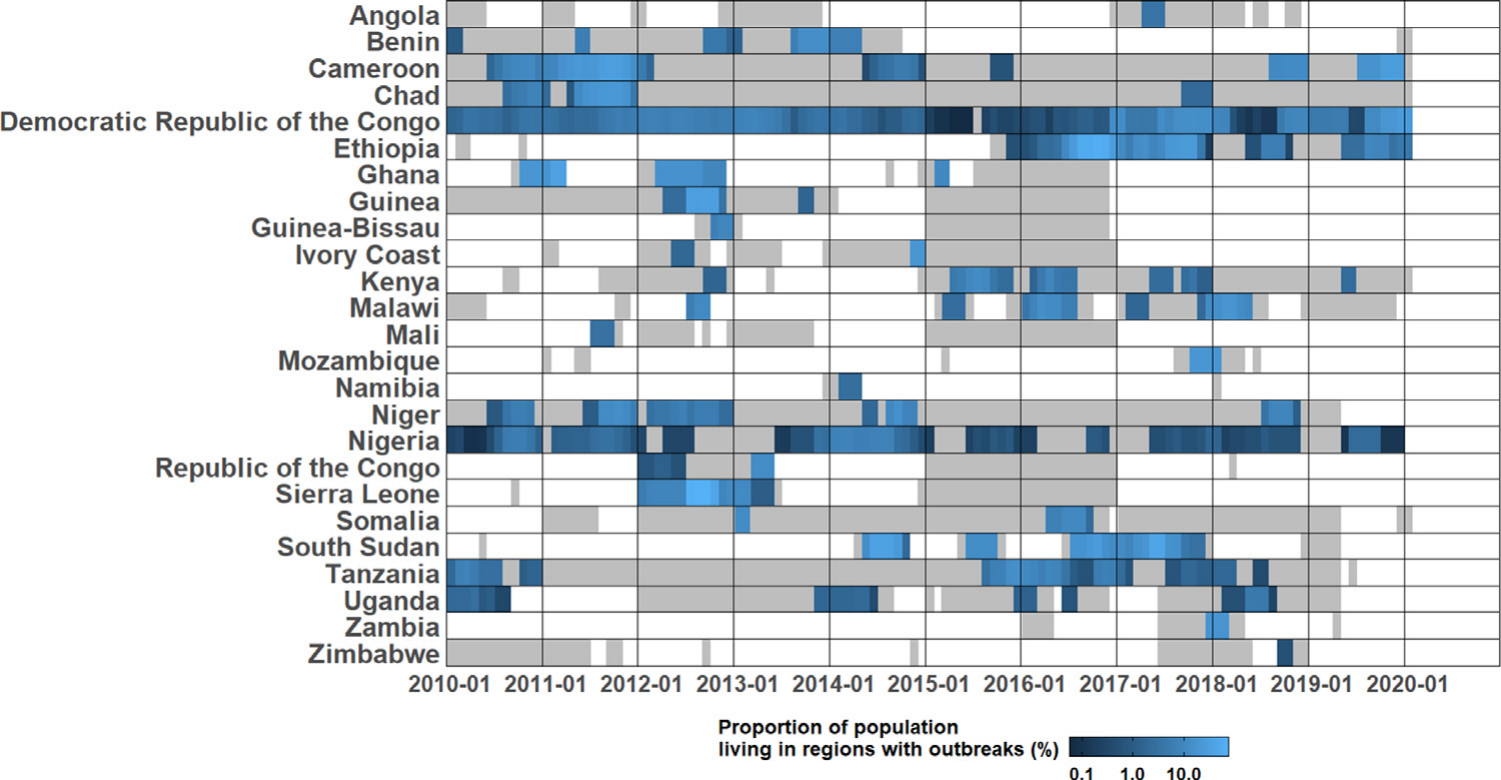
Proportion of population living in regions with outbreaks reported at the sub-national administrative units (%)**.** The proportion of population living in regions with outbreaks reported at sub-national administrative units for each month between January 1, 2010 and January 31, 2020. The areas in grey represent time periods covered by daily and weekly cholera reports. To combine the population at different spatial levels, we added the population of different regions together, and when an outbreak was reported at multiple spatial units, the population of the highest spatial unit was used to represent the population affected by that outbreak.

Over this 10-year period, 1.8 billion person-months (2% of the total during this period) were spent at risk of a cholera outbreak in sub-Saharan Africa (Figure 2 and Table S2), where all individuals living in a region with an outbreak were considered at risk. The collective outbreaks in four countries covered 65% of this total person-month burden: the Democratic Republic of the Congo (27.8%), Ethiopia (27.1%), Cameroon (6.3%), and South Sudan (3.2%). These values corresponded to 5% each of the Democratic Republic of the Congo and Ethiopia person-months, and 4% each of Cameroon and South Sudan person-months.

Outbreaks were extracted and examined by administrative levels, as reported by the original data source (Table 1). Across the 692 suspected case outbreaks reported in second-level administrative units, the median outbreak threshold was 0.7 per 100,000 people per week (interquartile range (IQR), 0.7-2.8 per 100,000 people per week), the median epidemic duration was 13 weeks (IQR, 8-19), the median time to outbreak peak was 4 weeks (IQR, 3-6), the median early outbreak reproductive number was 1.8 (range, 1.1-3.5), and the median attack rate was 0.8 per 1000 people (IQR, 0.3-2.4 per 1000 people) (Table 1 and Figures S3-7). One in four cases (median 23.8; IQR 16.4-34.5%) during each outbreak was reported in the week following the epidemic peak, with a median peak week incidence of 0.2 per 1000 people (IQR, 0.1-0.5 per 1000 people) (Table 1 and Figures S8-9).

**Table 1:** Summary of outbreaks of suspected cholera cases reported in sub-Saharan Africa, January 2010 to January 2020.

	Outbreaks reported at first-level administrative units	Outbreaks reported at second-level administrative units	Outbreaks reported at third-level administrative units
Metrics based on suspected cholera cases			
Number of outbreaks	62	692	245
Outbreak threshold,weekly incidence per 100,000 people (IQR)	0.7 (0.7-2.0)	0.7 (0.7-2.8)	1.7 (1.7-12.8)
Median outbreak size, cases (IQR)	620 (251-2,191)	182 (70-449)	100 (42-234)
Median epidemic durations, weeks (IQR)	12 (8-19)	13 (8-19)	12 (8-16)
Median time to epidemic peak, weeks (IQR)	4 (3-5.5)	4 (3-6)	3 (3-5)
Median proportion of suspected cases reported during the peak week (%) (IQR)	19.1 (14.8-29.7)	23.5 (16.4-34.2)	28.8 (20.5-38.1)
Median weekly incidence during the peak week per 1000 people (IQR)	0.1 (0.03-0.2)	0.2 (0.1-0.5)	0.5 (0.2-1.5)
Median early outbreak reproductive number (range)	1.8 (1.2-2.9)	1.8 (1.1-3.5)	1.9 (1.02-3.2)
Median attack rate per 1000 people (IQR)^b^	0.5 (0.2-1.2)	0.8 (0.3-2.4)	2.0 (0.6-6.4)
Metrics based on cholera-associated deaths^a^			
Number of outbreaks with reports of deaths	37	646	193
Median case fatality risk (%)(IQR)	1.2 (0.4-2.1)	1.6 (0.3-4)	0 (0-0.9)
Population-weighted case fatality risk (%)^c^	0.7	1.9	0.8

This table presents the key epidemic metrics of outbreaks by different administrative reporting units, including outbreak size, duration, time to outbreak peak, initial reproductive numbers during the first epidemic week, attack rate, and CFRs.

a N.B. Outbreaks with reports of deaths may not have documented this information systematically, so these results are highly sensitive to reporting biases.

b Only outbreaks with valid population estimates are included. There were 62 outbreaks at the first-level administrative units, 657 outbreaks at the second-level units, and 281 outbreaks at the third-level units.

c Only outbreaks with reports of deaths and valid population estimates are included. There were 36 outbreaks at the first-level administrative units, 610 outbreaks at the second-level units, and 229 outbreaks at the third-level units. CFR = case fatality risks; IQR = interquartile range.

Outbreaks reported at a higher spatial scale (e.g., province is higher than village) tended to have more suspected cases (e.g., median suspected cases of 620 vs 100 for first-level and third-level administrative units, respectively) but lower attack rates (e.g., median attack rate of 0.5 per 1000 vs 2 per 1000 for first-level and third-level, respectively) (Table 1 and Figures S7 and S10). Epidemic durations (e.g., median of 12-13 weeks across levels), times to peak (e.g., median of 4 weeks across levels), and early outbreak reproductive numbers were similar across spatial reporting units (e.g., median of 1.9-2 across levels) (Table 1 and Figures S4-6).

Only a subset of outbreaks also reported confirmed cases and deaths, and these data were not systematically reported (Table 1, Table S3, and Figures S11-14). For example, only 92 (13%) of second-level administrative unit outbreaks reported any confirmed case data (including zeros), with only 54 (8%) reported at least one confirmed cholera case; a median of 2.2% of suspected cases was confirmed (IQR, 0.5-9.8%) among outbreaks with at least one confirmed case (Table S3 and Figures S11-12). There were 646 (93%) outbreaks at second-level administrative units that reported cholera-associated deaths, with a median CFR of 1.6% (IQR, 0.3-4%) (Table 1 and Figures S13-14).

Outbreaks in rural and urban areas reported at a fine spatial-scale (3rd level administrative unit) had different characteristics. Urban outbreaks had higher peak weekly incidence (e.g., median peak weekly incidence of 0.4 versus 0.8 per 1000 people in rural and urban settings, respectively, p = 0.03) (Table 2 and Figure S22), greater attack rates (e.g., median attack rate per 1000 population of 1.5 versus 2.9 in rural and urban settings, respectively, p = 0.002) (Table 2 and Figure S23), longer epidemic durations (e.g., the median duration of 12 weeks versus 14 weeks in rural and urban settings, respectively, p = 0.03) (Table 2 and Figure S24), and a higher rate of confirmed cases per population (e.g., median confirmed cases per 1000 population of 0.04 versus 0.7 in rural and urban settings, respectively, p <0.001) (Table S5 and Figure S25). We found different results when comparing outbreaks reported at coarser spatial scales (Table S4). However, it is likely that many of the larger administrative units classified as rural were actually a mix of urban and rural areas, thus challenging the interpretation of these results.

**Table 2:** Outbreaks of suspected cholera cases reported at the third administrative level in rural and urban settings.

	Rural	Urban
Metrics based on suspected cholera cases		
Number of outbreaks	188	57
Outbreak threshold, weekly incidence per 100,000 population (IQR)	1.5^a^ (0.5-11.4)	3.0^a^ (1-28.2)
Median outbreak size (IQR)	100 (43-215)	96 (40-469)
Median epidemic durations, weeks (IQR)	12^a^ (8-15)	14^a^ (10-17)
Median time to epidemic peak, weeks (IQR)	3 (3-5)	4 (3-6)
Median proportion of suspected cases reported during the peak week (%) (IQR)	31.4^a^ (22.2-39.2)	22.7^a^ (16.7-30)
Median weekly incidence during the peak week per 1000 people (IQR)	0.4^a^ (0.1-1.4)	0.8^a^ (0.3-3)
Median early outbreak reproductive number (range)	1.9 (1.02-3)	2.0 (1.3-3.2)
Median attack rate per 1000 people (IQR)	1.5^a^ (0.6-5.7)	2.9^a^ (1.9-10.9)
Metrics based on cholera-associated deaths^b^		
Number of outbreaks with reports of deaths	138	55
Median case fatality risk (%)(IQR)	0 (0-1)	0 (0-0.2)

This table presents the comparisons of epidemic metrics between rural and urban settings for outbreaks at third-level administrative units.

a *P* <0.05 Wilcoxon rank sum test was used to test if the medians of outbreak characteristics are the same between rural and urban settings.

b N.B. Outbreaks with reports of deaths may not have documented this information systematically, so these results are highly sensitive to reporting biases. IQR= interquartile range.

**Figure 3:**
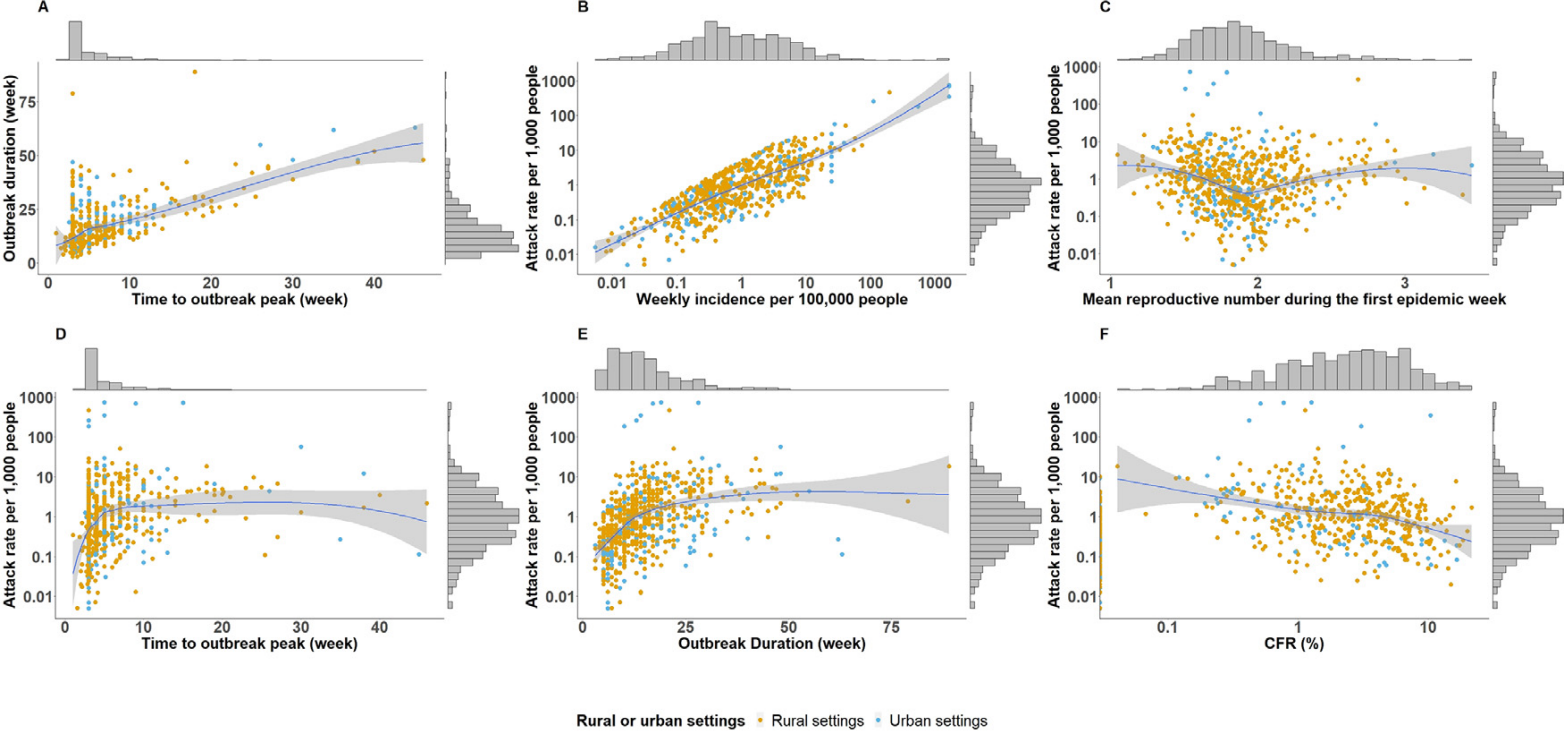
Bivariate relationships between epidemic metrics among outbreaks reported at the second-level administrative units. This figure shows the correlations between different epidemic metrics for second-level administrative unit outbreaks, including outbreak threshold, mean reproductive number during the first epidemic week, attack rate, duration, time to outbreak peak and CFR. The marginal histograms show the distributions of individual metrics (The attack rate, weekly incidence per 100,000 people, and CFR are in log scale, whereas other characteristics are in linear scale). Panel A shows the correlation between time to outbreak peak (week) and outbreak duration (week). Panel B shows the correlation between outbreak threshold (i.e., weekly incidence per 100,000 people) and attack rate per 1000 people. Panel C shows the correlation between mean reproductive number during the first epidemic week and attack rate per 1000 people. Panel D shows the correlation between time to outbreak peak (week) and attack rate per 1000 people. Panel E shows the correlation between outbreak duration (week) and attack rate per 1000 people. Panel F shows the correlation between CFR (%) and attack rate per 1000 people. CFR = case fatality risks.

We explored the relationships between outbreak metrics, focusing on outbreaks at the second administrative level (Figure 3, see Figures S26-27 for bivariate relationships at different spatial scales). Two comparisons, outbreak threshold versus attack rate (correlation = 0.75, *P*<0.0001), and time to outbreak peak versus epidemic duration had strong positive relationships (correlation = 0.62, *P* <0.0001). Larger attack rates were also associated with longer times to outbreak peak (correlation = 0.2, *P* <0.0001) and longer epidemics (correlation = 0.4, *P* <0.0001). Larger attack rates also appeared to have a negative relationship with CFRs (correlation = -0.3, *P* <0.0001). The early reproductive number did not appear to have an association with other outbreak metrics. These relationships were consistent across different spatial reporting units.

As a sensitivity analysis, we extracted outbreaks using an alternate outbreak threshold definition (see Methods) and found only minor changes to summary outbreak metrics (see Supplement and Tables S6-7 and Figures S39-74). We also examined outbreak metrics after grouping outbreaks by population size instead of administrative units (Table S8 and Figures S28-38). Case fatality risk increased monotonically for the three smaller population-sized groups and declined again for outbreaks in regions with populations greater than 1 million (Table S8 and Figure S28). Early reproductive numbers, outbreak duration, and time to outbreak peak were similar across regions with different population sizes (Table S8 and Figures S29-31).

## Discussion

We shed new light on cholera outbreaks in sub-Saharan Africa over the past decade by applying a systematic outbreak definition to time series from a large database of cholera incidence. We found that 2% or 1.8 billion person-months of the total sub-Saharan Africa population were at risk in regions with ongoing cholera outbreaks in the period from 2010 through 2019; this impact was spread across 999 suspected cholera outbreaks in 744 sub-national regions across 25 sub-Saharan African countries.

Timing (time to outbreak peak and epidemic duration) and incidence rate metrics (outbreak threshold and attack rate) had strong positive correlations with each other, and as may be expected, outbreaks with larger attack rates tended to be longer. Outbreaks with larger attack rates tended to have lower CFRs. The low specificity of suspected cholera case definitions and limited laboratory testing for cholera may mean that attack rates are overestimated, CFRs are underestimated, and that these metrics are negatively correlated.

Although a few studies report greater relative cholera burden and mortality in rural areas, high population density, and urban settings have long been thought to be drivers of *V. cholerae* in outbreaks (Cowman et al., 2017; Emilien, 2015; Mengel et al., 2014; Page et al., 2015; Penrose et al., 2010). Our results add nuance to this general wisdom in that population density may not be universally associated with more severe cholera outbreak outcomes; attack rates were higher, and epidemics were longer in urban settings for third-level administrative unit outbreaks, but the peak outbreak week captured a larger percentage of total outbreak cases in rural settings.

Summaries of historical outbreaks have served as the basis of planning for emergency cholera responses for decades, enabling organizations like MSF and UNICEF to perform data-driven allocation of resources like beds and rehydration fluids (Olson et al., 2018). Compared with existing planning resource tables, our results estimate that contemporary outbreaks in sub-Saharan Africa have much lower attack rates and CFRs in both urban and rural settings, a slightly higher proportion of cases reported during the peak week, and that peak bed capacity needs may be only one-third to one-fifth of existing estimates (Olson et al., 2018). Unlike the outbreak-specific surveillance used to develop these planning resource tables, our analyses used heterogeneous surveillance sources aggregated to administrative units, so these metrics may not be directly comparable.

This work may also support decision-making related to reactive oral cholera vaccine (OCV) campaigns, as our descriptive analysis may be used to inform prospective vaccine impact estimates for a given campaign. In addition, future policy guidance for emergency OCV requests may wish to leverage the positive association between outbreak incidence thresholds and overall outbreak attack rates (Figure 3 and Figures S26-27) to triage ongoing outbreak locations at greatest risk for a high attack rate.

Our analysis suggests that cholera outbreaks at larger geographic scales may be a compilation of outbreaks at smaller geographic scales because duration, peak timing, and early reproductive number metrics remained relatively stable across geographic scales (and for an alternate outbreak threshold definition, Table S4). The cholera incidence and mortality data were reported at the scale of administrative units (as opposed to the “outbreak” scale), which means that a single outbreak spanning multiple administrative units appears as multiple outbreaks, and outbreaks below the level of an administrative reporting unit would be aggregated to a higher scale. Our metrics are likely sensitive to reporting units, which vary greatly in size by country, and may not directly translate to outbreak metrics reported elsewhere, particularly for regions with smaller populations (see Table S8 for summary statistics by population size). However, third-level administrative units observed the highest outbreak thresholds, attack rates, and proportions of cases reported in the peak week (compared with larger geographic scales), and an assessment of outbreaks overlapping in space and time found that it was common for multiple third-level administrative units to report concurrent outbreaks at the same time as their surrounding second-level administrative unit (Figures S75-77). This suggests that multiple, geographically-proximate administrative level three units may often represent an epidemiologically-relevant geographic region for cholera outbreaks.

This work represents one of the most comprehensive analyses of cholera outbreaks, but reporting gaps between outbreaks posed challenges to outbreak extraction and may have biased our outbreak metrics; an average of 54% (range 5-100%) of the study period had some surveillance data coverage across all countries. Our analysis leverages but is also limited by its disparate data sources, case definitions, and testing and reporting protocols (Nadri et al., 2018). Cholera surveillance with laboratory confirmation is not collected or reported systematically across sub-Saharan Africa. Fewer than 13% of our outbreaks reported at least one confirmed case, likely because of limited reporting of laboratory data. Further, our outbreak threshold definition does not account for cholera seasonality, although sensitivity analysis suggests that our results are robust to alternate non-seasonal baseline definitions (Table S7-8) (Perez-Saez et al., 2022).

This study serves as an important baseline for monitoring progress toward cholera control in sub-Saharan Africa and provides essential information to inform cholera outbreak management and emergency response. As several countries have started to announce ambitious plans for large-scale reductions in cholera incidence and outbreak frequency, improvements in surveillance and reporting, including laboratory confirmation, will be key not only to describing the true burden but also to targeting interventions with scarce resources.

## Declaration of interests

The authors have no competing interests to declare.

## Funding

QZ, JL, ASA, and ECL were supported by the Bill and Melinda Gates Foundation (INV-002667).

## Ethical approval

Ethical approval was not required for this work.

## Appendix Supplementary materials

Image, application 1
